# Modulation of Reactive Oxygen Species Homeostasis as a Pleiotropic Effect of Commonly Used Drugs

**DOI:** 10.3389/fragi.2022.905261

**Published:** 2022-06-14

**Authors:** Carolin Thomas, Lia Wurzer, Ernst Malle, Michael Ristow, Corina T. Madreiter-Sokolowski

**Affiliations:** ^1^ Laboratory of Energy Metabolism Institute of Translational Medicine Department of Health Sciences and Technology ETH Zurich, Schwerzenbach, Switzerland; ^2^ Molecular Biology and Biochemistry, Gottfried Schatz Research Center, Medical University of Graz, Graz, Austria

**Keywords:** Aging, Reactive Oxygen Species, Mitohormesis, drugs, Pharmacology, pleiotropy

## Abstract

Age-associated diseases represent a growing burden for global health systems in our aging society. Consequently, we urgently need innovative strategies to counteract these pathological disturbances. Overwhelming generation of reactive oxygen species (ROS) is associated with age-related damage, leading to cellular dysfunction and, ultimately, diseases. However, low-dose ROS act as crucial signaling molecules and inducers of a vaccination-like response to boost antioxidant defense mechanisms, known as *mitohormesis*. Consequently, modulation of ROS homeostasis by nutrition, exercise, or pharmacological interventions is critical in aging. Numerous nutrients and approved drugs exhibit pleiotropic effects on ROS homeostasis. In the current review, we provide an overview of drugs affecting ROS generation and ROS detoxification and evaluate the potential of these effects to counteract the development and progression of age-related diseases. In case of inflammation-related dysfunctions, cardiovascular- and neurodegenerative diseases, it might be essential to strengthen antioxidant defense mechanisms in advance by low ROS level rises to boost the individual ROS defense mechanisms. In contrast, induction of overwhelming ROS production might be helpful to fight pathogens and kill cancer cells. While we outline the potential of ROS manipulation to counteract age-related dysfunction and diseases, we also raise the question about the proper intervention time and dosage.

## 1 Highlights

• Age-related diseases are associated with defective ROS homeostasis.
• Approved drugs exhibit pleiotropic effects on ROS homeostasis.
• Enforcement of antioxidant defense mechanisms positively affects cardiovascular and neurodegenerative diseases.
• Overwhelming ROS production might be used to fight pathogens and kill cancer cells.

## 2 Introduction

### 2.1 Reactive Oxygen Species Modulation as a Potential Treatment Strategy

The global number of elderly over age 80 will triple from 2015 until 2050. Since aging is associated with the progressive decline of functionality and regenerative potential of tissues, there is an urgent need to prolong a healthy lifespan and to find strategies to delay the onset of age-related dysfunctions such as chronic inflammation and pain, diabetes, cardiovascular diseases (CVD), neurodegenerative diseases, and cancer (Sun et al., 2010; Tejero et al., 2019). On a cellular level, dysfunctions in cellular signaling, proteostasis, autophagy, and mitochondrial homeostasis are closely intertwined in a vicious circle leading to loss of cellular homeostasis and health. For instance, poor protein quality control leads to defective organelles that produce enhanced levels of reactive oxygen species (ROS). These highly reactive chemicals are based on reduced molecular oxygen and include superoxide, hydroxyl radicals, singlet oxygen, and peroxides (Sun et al., 2010; Tejero et al., 2019). In addition to superoxide, nitric oxide synthases (NOS) also produce nitric oxide that interacts with ROS to rapidly produce reactive nitrogen species (RNS) such as peroxynitrite (Sun et al., 2010; Tejero et al., 2019). Due to the electron transport chain (ETC), mitochondria are the cell’s main production sites of ROS (Sun et al., 2010; Tejero et al., 2019). Notably, a reduction in mitochondrial content was found to counteract the process of aging *in vivo* (Correia-Melo et al., 2016), suggesting that mitochondrial activity gets detrimental during aging. Besides, an enormous number of more than 200 clinical disorders, including type 2 diabetes mellitus (T2DM), degenerative brain impairments like Alzheimer’s disease (AD) and Parkinson’s disease (PD), cardiovascular dysfunction, and cancer, have been associated with early dysregulations in redox homeostasis through so-called ROS and NOS (Kaul et al., 2001; Farah et al., 2018). Besides, experiments in various species revealed an inverse (Schulz et al., 2007; Singh et al., 2011; Ristow and Schmeisser, 2014; Cai et al., 2017) correlation between ROS production rates and lifespan (Sun et al., 2010; Tejero et al., 2019). Align with these findings, increased formation of mitochondrial ROS (mtROS) was postulated as the primary cause of aging in (Harman, 1956) in the *Free Radical Theory of Aging* (Sun et al., 2010; Tejero et al., 2019). Consequently, in the hope of successfully counteracting age-related diseases, numerous clinical trials tested the impact of antioxidants, including natural or artificial ROS scavenging substances, on the development or progression of age-related diseases. Unfortunately, most clinical trials failed to reveal a benefit from the use of antioxidants or were even related to harmful side-effects on human health like cancer growth. For instance, a randomized controlled trial found that supplementation with vitamin E over 7 years does not prevent cancer or major cardiovascular events but increases the risk for heart failure in patients with vascular diseases or diabetes mellitus (Sun et al., 2010; Tejero et al., 2019). While the application of antioxidants failed to prove a beneficial effect in clinical trials, interventions boosting the body’s antioxidant defense mechanisms are associated with enhanced health and lifespan in various species. A transient ROS burst functions like a vaccine, thereby enabling an adaptational response with enhanced antioxidant defense mechanisms. Notably, behavioral interventions like caloric restriction or physical activity, both known to positively affect health and lifespan, as well as compounds like green tea catechins, long time associated with healthy aging, might trigger these vaccination-like ROS level rises (Merry and Ristow, 2016), (Schulz et al., 2007; Singh et al., 2011; Ristow and Schmeisser, 2014; Cai et al., 2017). Based on these reports, it might be essential to strengthen antioxidant defense mechanisms in advance by low ROS level rises to maintain a proper ROS homeostasis during age and to prevent age-related diseases (Ristow and Schmeisser, 2014). It remains questionable whether selectively targeting certain ROS species within the cell might be an intervention strategy when lacking the potential to induce antioxidant defense mechanisms, for instance, in the case of amyotrophic lateral sclerosis (ALS) (Carrera-Juliá et al., 2020). In contrast, overwhelming ROS production might also be desirable under certain conditions, including fighting off pathogens (Paiva and Bozza, 2014) or cancerous cells (Liou and Storz, 2010).

A strategy to delay the onset of age-related diseases by ROS modulation might require drug administration to still healthy and comparably young individuals for extended periods. Consequently, respective drug candidates are needed to be safe and exhibit a minimum of side effects in the long-term use. To identify drugs least likely to cause harm while still providing benefit, numerous classes of potential geroprotective compounds are currently tested in model organisms like *Saccharomyces cerevisiae*, *Caenorhabditis elegans*, and *Drosophila melanogaster* (Janssens and Houtkooper, 2020). According to the U.S. Food and Drug Administration (FDA), an experimental drug takes 12 years on average to progress from bench to market. Usually, only 5 out of 5000 pre-clinically tested compounds will be used in clinical trials, and only one out of these five clinically tested compounds will receive approval for therapeutic use (Kraljevic et al., 2004). Consequently, it might be worthwhile to evaluate whether approved drugs that are already characterized regarding their long-term safety and side effects might be repurposed to modulate ROS homeostasis. Notably, several drugs in clinical use exhibited pleiotropic antioxidative effects and were found to delay aging in various aging models (Steven et al., 2015; Janssens and Houtkooper, 2020). The current review provides an up-to-date overview of how approved drugs interfere with ROS homeostasis and evaluates their potential to counteract the age-related decline in cellular function.

### 2.2 Molecular targets

#### 2.2.1 ROS production sites

##### 2.2.1.1 Mitochondrial

Mitochondria are the main production site of ROS (Boveris et al., 1972). Superoxide and hydrogen peroxide are generated as a side product of mitochondrial respiration during electron leakage at the respiratory chain complexes I, II, and III in the inner mitochondrial membrane (Goncalves et al., 2015) ( Table 1). Since excessive mtROS production is associated with age-related dysfunction and pathologies, including tumor growth (Bell et al., 2011) and hypertension (Kimura et al., 2005; Dikalova et al., 2010), mtROS was long time seen as a harmful by-product of respiration only. However, moderate levels of mtROS are crucial for various signaling pathways (Collins et al., 2012), such as response to hypoxia (Guzy and Schumacker, 2006), cell differentiation (Mandal et al., 2011), autophagy (Lee et al., 2012a), inflammation (Zhou et al., 2011), and immune response (West et al., 2011). Notably, mitochondrial damage-associated molecular patterns (mtDAMP), which are indicators of mitochondrial dysfunction, play a critical role in ROS-mediated inflammatory processes. For example, oxidated mtDAMPs such as mitochondrial DNA (mtDNA) were shown to activate the NLR family pyrin domain containing 3 (NLRP3) inflammasome, an essential component of the innate immune system (Shimada et al., 2012; Zhong et al., 2013).

**TABLE 1:** Source and localization of reactive oxygen species (ROS).

Molecule	Oxidant formed	Enzymes	Localization
Molecular oxygen (O_2_)	Superoxide (O_2_^−^)	NOX1-5, DUOX1-2	Plasma membrane and intracellular membranes
		XO, XDH	Cytoplasm
		ETC	Inner mitochondrial membrane
Superoxide (O_2_^−^)	Hydrogen Peroxide (H_2_O_2_)	SOD1	Cytoplasm, peroxisomes, lysosomes, nucleus
		SOD2	Mitochondrial matrix
		SOD3	Extracellular space
Molecular oxygen (O_2_)	Hydrogen Peroxide (H_2_O_2_)	MAO	Outer mitochondrial membrane

Monoamine oxidase (MAO): The mitochondrial monoamine oxidases (MAOs) A and B degrade a variety of neurotransmitters, including norepinephrine, serotonin, dopamine, and tyramine (Sub Laban and Saadabadi, 2021), by catalyzing the oxidative deamination of their amines (Tipton, 2018). Thereby, hydrogen peroxide is generated as a side product at the outer mitochondrial membrane that possibly contributes to oxidative stress (Table 1). Nonetheless, there is no clinical proof that MAO inhibitors reduce levels of toxic and prooxidant MAO products until now (Tipton, 2018). In the clinic, selective MAO-A inhibitors are used as amine-depleting drugs against depression (Kline, 1958; Zeller et al., 1959). In contrast, selective MAO-B inhibitors work as dopamine-sparing compounds against PD (selective MAO-B inhibitors) (Birkmayer et al., 1983).

##### 2.2.1.2 Non-Mitochondrial

NADPH oxidase 1–7: NADPH oxidases (NOXs) are transmembrane enzymes that specifically generate radical superoxide anions by reducing molecular oxygen using nicotinamide adenine dinucleotide phosphate (NADPH) (Table 1). The NOX family consists of 7 members, including NOX1-5, as well as the dual oxidases 1 and 2 (DUOX1; DUOX2), which are expressed in endothelial cells, vascular smooth muscle cells, cardiac myocytes and fibroblasts, adipocytes, macrophages, stem cells, and adventitial fibroblasts (Lassègue et al., 2012). Although the prooxidant role of NOXs is crucial in cellular physiology, such as differentiation, proliferation, apoptosis, inflammatory responses, host defense, and redox signaling (Lassègue et al., 2012; Vermot et al., 2021), increased NOX activity was also correlated with several pathologies such as neurodegenerative diseases, including AD and PD (Sorce and Krause, 2009; Popa-Wagner et al., 2013; Vermot et al., 2021) and cancer (Juhasz et al., 2009; Meitzler et al., 2014; Wang et al., 2015; You et al., 2018). Targeting NOXs might therefore represent a suitable approach for disease-specific therapies (Spencer and Engelhardt, 2014).

Xanthine oxidoreductase: The xanthine oxidoreductase (XOR) belongs to the family of molybdoenzymes (Kisker et al., 1997) and is expressed in human tissues such as the liver, small intestine, mammary gland (Linder et al., 1999), and heart (Muxfeldt and Schaper, 1987; Abadeh et al., 1993; Vickers et al., 1998). XOR can be interconverted into xanthine dehydrogenase (XDH) and xanthine oxidase (XO) (Stirpe et al., 1969). Both XDH and XO are involved in the metabolism of hypoxanthine/xanthine to uric acid during purine degradation (Xu et al., 1996; Okamoto et al., 2013) upon generation of superoxide anion (Battelli et al., 2016) (Table 1). Besides the involvement of XOR in purine catabolism, there is evidence for a broader range of benefits. For example, it was speculated that XOR-derived uric acid positively impacts oxidative stress-associated aging and cancer and thus promotes the extension of life span in humans (Ames et al., 1981).

Nitric oxide synthase (NOS): The family of nitric oxide synthases includes neuronal NOS (nNOS), inducible NOS (iNOS), and endothelial NOS (eNOS). These enzymes generate nitric oxide upon L-arginine utilization which plays an essential part in vascular regulation, inflammation, and intracellular signaling. Besides these physiological functions, nitric oxide also represents a harmful contributor to oxidative stress as it can react to cytotoxic peroxynitrite in the presence of oxygen (Adams et al., 2015).

#### 2.2.2 The Antioxidant System

The cellular antioxidant system is subdivided into enzymatic antioxidants, including the glutathione and thioredoxin system, and nonenzymatic antioxidants, such as dietary vitamins. Polymorphisms in involved proteins such as superoxide dismutase (SOD), catalase (CAT), and glutathione peroxidase (GPX) were associated with various human metabolic disorders such as diabetes, CVD, and cancer (Crawford et al., 2012; Hebert-Schuster et al., 2012), emphasizing the critical role of enzymatic antioxidant systems.

Superoxide dismutase 1–3: The family of superoxide dismutases (SODs) consists of the copper-zinc superoxide dismutase (*SOD1* gene) localized in the cytoplasm, peroxisomes, lysosomes, and the nucleus (Chang et al., 1988; Keller et al., 1991; Crapo et al., 1992; Liou et al., 1993), as well as the manganese superoxide dismutase (*SOD2* gene) present in the mitochondrial matrix (Weisiger and Fridovich, 1973), and the extracellular superoxide dismutase (*SOD3* gene) (Marklund, 1984). SODs are the only class of enzymes catalyzing the reduction of superoxide to hydrogen peroxide and O_2_ (Genestra, 2007) and thus playing a significant role in redox regulation (Table 1). Malfunction of SOD is associated with several clinical pathologies. For example, *SOD1* mutations were able to mimic ALS-associated dysregulation in several mouse models (Bruijn et al., 2004). In addition, *SOD2* mutations are associated with cardiomyopathy, sporadic motor neuron defects, and cancer (Hiroi et al., 1999). On the other hand, overexpression of SOD1 proved beneficial against neurological injuries and brain diseases in animal studies (Kamii et al., 1999; Morita-Fujimura et al., 2000; Sugawara et al., 2002; Yu et al., 2006; Endo et al., 2007).

Catalase: Human CAT is a peroxisomal enzyme that detoxifies hydrogen peroxide by catalyzing its reduction to H_2_O and O_2_ (Putnam et al., 2000). Overexpression of catalases in mitochondria results in extended life span with attenuated age-related pathologies (Schriner et al., 2005; Treuting et al., 2008; Dai et al., 2009; Olsen et al., 2013) and protects against CVD (Yang et al., 2003; Yang et al., 2009; Maiellaro-Rafferty et al., 2011) and diabetic nephropathy (Brezniceanu et al., 2007; Shi et al., 2013) in mice. In contrast, patients that lack catalase expression are prone to develop T2DM (Avraham et al., 1988).

Thioredoxin system: The thioredoxin system involves several players and types of redox reactions. In first-line are peroxiredoxins (PRX1-6), a family of peroxidases (Wood et al., 2003; Flohé et al., 2011; Rhee and Woo, 2011) that catalyze the reduction of hydrogen peroxide by oxidation of their active PRX disulfides. Regeneration of oxidized PRX is achieved by reduction through small thiol-disulfide oxidoreductases, so-called thioredoxins (TRX). Thioredoxins are eventually reduced by thioredoxin reductases (TR) upon utilization of the reductive potential of reduced NADPH (Lillig and Holmgren, 2007; Lu and Holmgren, 2014). Strengthening the thioredoxin system manifests in several health-beneficial alterations. For example, overexpression of PRX3 reduced cardiac failure upon myocardial infarction in murine heart mitochondria (Matsushima et al., 2006) and counteracted hyperglycemia and glucose intolerance of mice (Chen et al., 2008). TRX1 was detected in the plasma during inflammation and oxidative stress (Nakamura et al., 1996) and proposed as antioxidant therapy (Nakamura et al., 2009; Watanabe et al., 2010; Matsuo and Yodoi, 2013). Moreover, TRX1 and TRX80 displayed protective effects in AD in humans (Gil-Bea et al., 2012).

Glutathione system: Glutathione peroxidases (GPXs) use a reducing equivalence of glutathione (GSH) to reduce peroxides and hydroxy radicals into nontoxic derivatives (Brigelius-Flohé, 1999; Flohé et al., 2011). The resulting oxidized glutathione (GSSG) is reduced to GSH by glutathione reductase (GR) through the oxidation of NADPH to NADP+ (Birben et al., 2012). Another representative of the glutathione system is the class of glutaredoxins (GRXs), which are oxidoreductases mainly responsible for the reduction of GSH-disulfides (Holmgren, 1976) and deglutathionylation of S-glutathionylated proteins (Holmgren et al., 2005). The glutathione system is the principal regulator of the cellular redox balance, and the GSH/GSSG ratio is used as an oxidative stress biomarker for several diseases, including CVD, AD, PD, ALS, multiple sclerosis (MS), and cancer (Frijhoff et al., 2015). GPX1 knock-out increases oxidative stress and the prevalence of AD-associated neurotoxicity and heart ischemia-reperfusion injury in mice (Yoshida et al., 1997; Fu et al., 1999; Klivenyi et al., 2000; Crack et al., 2006; Lim et al., 2009). In contrast, GPX1 overexpression protected against oxidative stress, cerebral ischemia/reperfusion damage, and neurodegenerative pathologies such as PD (Weisbrot-Lefkowitz et al., 1998; Sheldon et al., 2004; Ridet et al., 2006). Several clinical trials linked polymorphisms of GPX and consequently decreased enzyme activity to an increased risk for T2DM (Ramprasath et al., 2012), cardiovascular dysregulations in T2DM patients (Hamanishi et al., 2004), breast cancer (Ravn-Haren et al., 2006), and colorectal adenomas (Hansen et al., 2005).

Nonenzymatic antioxidants: Nonenzymatic antioxidants include low molecular mass molecules such as carotenoids, vitamin A, ascorbic acid (vitamin C), α-tocopherol (vitamin E), polyphenols, minerals such as selenium and zinc, as well as various drugs including acetylcysteine (Pisoschi and Pop, 2015). The antioxidant potential of vitamin A, C, and E, as well as phenolic acids, is given by their ability to scavenge free radicals in the form of ROS and RNS (Burton and Ingold, 1984; Burton and Traber, 1990). In addition, carotenoids and vitamin E prevent lipid peroxidation by direct reduction of peroxyl radicals (Burton and Ingold, 1984). Minerals, instead, are crucial components of antioxidant enzymes and consequently crucial for their activity (Tabassum et al., 2010). Zinc, for example, is an inhibitor of NADPH oxidase and a part of superoxide dismutase and thus contributes to the antioxidant system. Although more than 100 clinical trials tested nutritional interventions with vitamins, polyphenols, and minerals have been conducted during the last twodecades (Bjelakovic et al., 2007; Bjelakovic et al., 2012), the vast majority failed to reveal the beneficial effects of dietary antioxidant supplementation (Goodman et al., 2011; Bjelakovic et al., 2012; Halliwell, 2013), questioning the total suppression of ROS generation as a therapeutical approach.

## 3 The implication of Reactive Oxygen Species in Age-Related Diseases

### 3.1 Cardiovascular Diseases

#### 3.1.1 Clinical Significance

Cardiovascular diseases (CVD) like ischemic heart diseases and stroke are the leading disease burden worldwide, causing the majority of cases in global mortality and contributing to various disabilities. There has been a worrying trend, with cases of CVD roughly doubling from 1990 to 2019, reaching 18.6 million (Roth et al., 2020). Deterioration of ROS homeostasis is a common hallmark in CVD, and ROS-modified molecules might even serve as biomarkers for the progression of CVD. For instance, clinical trials have revealed an association between increased levels of circulating oxidative low-density lipoprotein (LDL) and atherosclerotic CVD (Gao and Liu, 2017). Moreover, 8-hydroxy-2-deoxyguanosine, a marker for oxidative DNA damage, is significantly increased in the serum patients with dilated cardiomyopathy (Kono et al., 2006), and the concentration in the urine correlates with heart failure (Kobayashi et al., 2011). Besides, 8-iso-prostaglandin F2α, a by-product of lipid peroxides generated during oxidative stress, is increased in patients with symptomatic heart failure and correlated with the functional severity of heart failure (Mallat et al., 1998). In contrast, another primary lipid peroxidation product, 4-hydroxy-2-nonenal-modified protein, was found to be elevated in the myocardium of hypertrophic cardiomyopathy patients (Nakamura et al., 2005). Despite the strong implication of ROS in CVD, clinical trials have failed to provide evidence for a therapeutic benefit of potent antioxidants in treating CVD so far (Panth et al., 2016), potentially due to blocking signaling function of ROS and by preventing ROS-induced upregulation of ROS defense mechanisms.

#### 3.1.2 Cellular Mechanisms and Signaling

ROS have a crucial signaling function in the cardiovascular system. For instance, ROS contribute to the signal transduction pathway of angiotensin II, causing cardiac growth and hypertrophy in neonatal rat cardiomyocytes (Shih et al., 2001). Moreover, ROS adjust the iron homeostasis in response to catecholamines in cardiomyocytes, a process essential to maintaining proper metabolic activity (Costa et al., 2009). Notably, enhanced ROS production is associated with left ventricular hypertrophy, and heart failure in experimental guinea pig models with left ventricular hypertrophy exhibited a progressive expression increase in several NADPH oxidase subunits (Li et al., 2002). Notably, the expression of NOX2 was also found to be increased in infarcted areas but unchanged in unaffected regions of cardiac samples from patients who had died from acute myocardial infarction (Krijnen et al., 2003). Platelet-derived ROS function as signaling molecules but might induce a vicious circle resulting in a platelet procoagulant phenotype and apoptosis, enhancing the thrombotic risk (Masselli et al., 2020). Besides, a deterioration of ROS defense mechanisms might contribute to the genesis and progression of CVD. Experiments in a cardiomyocyte-specific SOD2 deficient mouse strain revealed that deficiency of SOD2 results in increased ROS levels and subsequent overproduction of electrophilic aldehydes, which serve as mediators of mitochondrial dysfunction and boost cardiomyopathy (Sharma et al., 2020). Besides, attenuated SOD2 activity resulted in enhanced mitochondrial oxidative stress and plaque instability in hyperlipidemic mice during aging (Vendrov et al., 2017). Enhanced ROS production and decreased ROS detoxification might also facilitate the formation of peroxynitrite that harms the vascular endothelium, smooth muscle, and myocardium (Pacher and Szabo, 2006). Besides, peroxynitrite formation reduces the amount of available nitric oxide that inhibits platelet activation and aggregation, cell adhesion molecule expression, and vascular smooth muscle proliferation and is, therefore, a crucial vasoprotective substance (Naseem, 2005).

### 3.2 Type 2 Diabetes Mellitus

#### 3.2.1 Clinical Significance

Type 2 diabetes mellitus (T2DM) is characterized by insufficient insulin secretion, an uncontrolled rise in blood glucose levels, and insulin resistance of peripheral tissues (Rochette et al., 2014). Besides genetic components and age, obesity, diminished physical activity, chronic inflammation, and elevated cholesterol and triglyceride levels are the main primary risk factors of T2DM (Zeller et al., 2008; Olsson et al., 2011; Verdile et al., 2015), emphasizing the importance of functional metabolic regulations. An untreated T2DM condition and prolonged periods of high blood glucose may cause damage to the vascular system, subsequently leading to stroke, peripheral vascular diseases, neuropathy, retinopathy, and nephropathy (Wallace and Matthews, 2004; Asmat et al., 2016). Notably, the severity and T2DM-related mortality are linked to vascular complications and positively correlate with the degree of oxidative stress and ROS (Andreev and Rybakov, 1975; Domingueti et al., 2016). Cellular damage was associated with a ROS-dependent activation of stress pathways such as nuclear factor kappa-light-chain-enhancer of activated B cells (NF-kB), c-jun N-terminal kinase (JNK)/stress-activated phosphor-kinase (SAPK), and p38 MAPK in T2DM with vascular complications (Evans et al., 2003). Other publications could measure elevated levels of NADPH and superoxide during vascular dysfunction in diabetic patients (Guzik et al., 2002; Ergul et al., 2005). Further evidence of increased ROS levels manifests in increased lipid peroxidation (Davì et al., 1999; Bandeira et al., 2012) and carbonylation of serum proteins of type 2 diabetes mellitus individuals (Pandey et al., 2010; Gradinaru et al., 2013). Besides, oxidative markers such as F2-isoprostane, nitrotyrosine (Ceriello et al., 2001; Odegaard et al., 2016), and glycated hemoglobin (HbA1c) (Ryden et al., 2013) are commonly found in plasma, urine, and tissues of T2DM patients and serve as biomarkers for hyperglycemia.

#### 3.2.2 Cellular Mechanisms and Signaling

ROS signaling plays a significant role in the metabolic activity of pancreatic β-cells (Ahmed Alfar et al., 2017), as well as downstream insulin signaling and glucose uptake in adipocytes by activation of the phosphoinositol 3-kinase and protein kinase B (AKT) (Mahadev et al., 2001), most likely mediated by NOX4-dependent ROS generation (Mahadev et al., 2004). Thereby, the high ROS sensitivity of pancreatic β cells is crucial for a functional physiological metabolism, as well as β cell regeneration and proliferation (Ahmed Alfar et al., 2017; Wang and Wang, 2017). However, the comparably low levels of antioxidant proteins such as GPX, CAT, SOD, and TR (Evans et al., 2003; Newsholme et al., 2007), make β-cells highly vulnerable to oxidative stress and ROS overload provoked by stimulation of the respiratory chain activity or NOX activity (Newsholme et al., 2007; Newsholme et al., 2012). Chronic oxidative stress impairs the cellular function of β-cells. It results in apoptotic cell death *via* p38 MAPK, JNK, and NF-kB signaling (Heimberg et al., 2001; Gurzov and Eizirik, 2011), reducing insulin secretion and provoking hyperglycemia. In turn, hyperglycemia excessively generates electron donors in the tricarboxylic acid (TCA) cycle and thus, promotes hyperpolarization of the mitochondrial membrane potential (*Ψ*_mito_) and adenosine triphosphate (ATP) generation, followed by inhibition of complex III of the respiratory chain and electron accumulation at coenzyme Q. Consequently, oxygen is only partially reduced, boosting the production of superoxide radicals (Korshunov et al., 1997; Nishikawa et al., 2000; Brownlee, 2001). In addition, inflammation-associated ROS was shown to inhibit insulin receptor activity, the respective signaling, and consequently the response to insulin (Newsholme et al., 2014; Verdile et al., 2015), eventually leading to insulin resistance. Furthermore, it was shown that vascular homeostasis and anti-inflammatory processes are impaired in diabetes due to poor production of nitric oxide (Shi and Vanhoutte, 2017), potentially due to enhanced peroxynitrite formation (Caldwell et al., 2015). The interplay of poor NO levels, increased peroxynitrite concentrations, and elevated production of ROS does consequently further increase oxidative stress in diabetes (Pacher and Szabo, 2006).

### 3.3 Neurodegenerative Diseases

#### 3.3.1 Clinical Significance

Alzheimer’s disease (AD) is a neurodegenerative disorder characterized by neuroinflammation, synaptic disruption, and abnormalities in mitochondrial structure and function (Hirai et al., 2001; Selkoe, 2002; Swerdlow and Khan, 2004; Barsoum et al., 2006; Wyss-Coray, 2006; Swerdlow et al., 2010), resulting in loss of memory and cognitive decline (Bertoni-Freddari et al., 1990; DeKosky et al., 1996; Tampellini and Gouras, 2010). (Walsh and Selkoe, 2007). As a consequence of an increasing global life expectancy (Jaul and Barron, 2017) and due to a growing population, the number of patients suffering from AD is predicted to increase in the future dramatically. While 35.6 million people were diagnosed with dementia in 2010, this number is estimated to reach 115.4 million people by 2050 (Prince et al., 2013) and thus strongly impacts worldwide health systems. One hallmark of AD is amyloid-β (Aβ) plaque formation due to abnormal processing of the amyloid precursor protein. Under pathological conditions, the amyloid precursor protein is cleaved into Aβ fragments (LaFerla et al., 2007), which oligomerize into soluble aggregates and subsequently accumulate to insoluble and toxic Aβ plaques. Consequently, oxidative damage of proteins, DNA, RNA, and lipids was found in AD patients’ brains (Reddy, 2006; Chaturvedi and Beal, 2008; Reddy and Beal, 2008). Thereby, oxidation productions such as 8-hydroxyguanosine and heme oxygenase serve as diagnostic markers in AD patients (Chaturvedi and Beal, 2008). Another important marker for AD is nitrotyrosine, a product of protein oxidation *via* nitric oxide (Good et al., 1996; Smith et al., 1997). Moreover, peroxynitrite production seems to be responsible for lysine, arginine, proline, and histidine oxidation in AD patients (Stadtman, 1990; Smith et al., 1997). Dysregulations in AD are undoubtedly associated with an imbalance in ROS homeostasis and subsequent oxidative damage in the brain of aged organisms (Migliaccio et al., 1999; Giorgio et al., 2005; Calkins et al., 2012). However, the molecular mechanisms are still elusive.

Parkinson’s disease (PD) is the fastest-growing neurodegenerative disease (Collaborators GBDN, 2019), and the number of people affected is predicted to double by 2040 (Dorsey and Bloem, 2018). PD is characterized by a progressive loss of dopaminergic neurons in the brain region substantia nigra (SN), leading to motor deficiencies such as tremor, rigidity, and bradykinesia. Another hallmark of PD is the development of insoluble inclusions consisting of aggregated α-synucleins, so-called Lewy bodies (Spillantini et al., 1997; Bellucci et al., 2012; Bellucci et al., 2016). While monomeric and tetrameric α-synucleins fulfill their physiological function as transport molecules, the formation of oligomers and fibrils is caused by α-synuclein mutation and contributes to pathological dysregulations (Bartels et al., 2011; Marques and Outeiro, 2012). Besides mutations in α-synuclein, familiar forms of PD might exhibit mutations in PTEN-induced kinase 1 (PINK) and E3 ubiquitin ligase (PARKIN), two proteins involved in autophagy (Lazarou et al., 2015; Pickrell and Youle, 2015), Parkinson’s disease protein 1 (PARK7 a redox-chaperone acting as oxidative stress sensor (Canet-Avilés et al., 2004), (Zondler et al., 2014), and leucine-rich repeat kinase 2 (LRRK2). Notably, these proteins are relevant for mitochondrial function and ROS homeostasis (Polymeropoulos et al., 1997; Bonifati et al., 2003; Valente et al., 2004; Gilks et al., 2005; Nichols et al., 2005). Indeed, the accumulation of damaged mitochondria is a further determinant for the mediation of PD pathology (Koentjoro et al., 2017). Neurons in the SN are highly vulnerable to oxidative stress and neurodegeneration, as seen in healthy elderly brains that contain double the number of oxidized proteins compared to other brain regions (Floor and Wetzel, 1998). Additionally, postmortem analyses of healthy aged brains compared to young controls revealed a decrease in SOD, GPX, and glutathione reductase activities (Venkateshappa et al., 2012), suggesting age-related loss of antioxidant enzyme activity. Another source of ROS represents the oxidative metabolism of dopamine, as hydrogen peroxide is produced as a side product of oxidative deamination of dopamine by MAO (Goldstein et al., 2013; Meiser et al., 2013). Furthermore, PD brains exhibit iron-enriched neurons in the SN (Dexter et al., 1987; Dexter et al., 1989; Michaeli et al., 2007; Pyatigorskaya et al., 2015), making this brain region highly vulnerable to oxidative stress (Brian J. Tabner et al., 2001; Jellen et al., 2013). Together with dopamine, labile iron is part of a prooxidant synergic interplay in aged SN (Hare and Double, 2016), leading to increased production of highly toxic dopamine-o-quinones (Tse et al., 1976; Graham, 1978; Zhou et al., 2010). The dopamine-o-quinones derivatives tetrahydroisoquinoline salsolinol and 6-hydroxydopamine were shown to increase ROS and oxidative stress by impairing ETC function (Su et al., 2013; Puspita et al., 2017). Chelation of iron as a treatment for PD was successful in animal models (Devos et al., 2014) and is currently tested in patients with early-stage PD by FAIRPARKII (ClinicalTrials.gov Identifier: NCT02655315).

#### 3.3.2 Cellular Mechanisms and Signaling

Alzheimer’s disease (AD): Neuronal function is strongly dependent on a high energy supply. Based on calculations, neurons use up to 50% of their ATP for homeostasis and re-establishment of the ion gradient and 30% for synaptic transmission (Ames, 2000; Attwell and Laughlin, 2001). Consequently, neurons strongly rely on oxygen availability and functional mitochondrial oxidative phosphorylation for ATP generation. However, high oxygen levels and enhanced mitochondrial activity provoke the formation of ROS (Zorov et al., 2014). In addition, neurons contain elevated levels of lipids and comparably low amounts of antioxidant enzymes, making them highly vulnerable to oxidative stress (Jang et al., 2010). Also, the synaptic transmission that includes vesicle formation is energy-intense (Attwell and Laughlin, 2001). Therefore, ATP-generating mitochondria need to undergo axonal transport from the soma to the distant periphery to supply synapses with sufficient energy. Notably, impairment of this process was found in a range of neurodegenerative diseases such as ALS, Huntington’s disease, AD, and PD (Martin, 2011; Schon and Przedborski, 2011; Reddy and Shirendeb, 2012), once again emphasizing the importance of mitochondria for neuronal function. Several authors suggest that lack of mitochondrial transport represents an early event during neurodegeneration and the pathology of AD (Rui et al., 2006; Wang et al., 2010a; Du et al., 2010; Calkins and Reddy, 2011). Importantly, dysfunctional mitochondrial transport, enormous mitochondrial fragmentation, attenuated synaptic ATP, and synaptic dysfunction in AD neurons were associated with increased oxidative stress (Reddy et al., 2012) (Reddy, 2006). Similar observations were found in the brains of AD patients, which exhibited attenuated ATP levels, elevated levels of free radicals, OXPHOS disruptions, and mitochondrial dysfunctions (Gibson et al., 1998; Maurer et al., 2000; Wang et al., 2005; Devi et al., 2006; Wang et al., 2008; Reddy, 2009; Reddy et al., 2010).

Parkinson’s disease (PD): The susceptibility towards oxidative damage is based on several specific features of dopaminergic neurons. *First*, neurons of the SN are comparably large and unmyelinated (Pissadaki and Bolam, 2013), resulting in high demand for ATP to maintain the *Ψ*_mito_, action potential, and synaptic transmission. *Secondly*, SN dopaminergic neurons generate constant action potentials autonomously without dependency on synaptic input to maintain dopamine levels in surrounding brain regions (Grace and Bunney, 1983; Romo and Schultz, 1990). *Thirdly*, dopaminergic neurons are Ca^2+^ pacemakers with constant buffering activity (Olson et al., 2005). Increased Ca^2+^ levels might boost ETC activity leading to a pathological increase in ROS and the initiation of apoptotic pathways (Joza et al., 2001; Malhotra and Kaufman, 2007). In summary, all these processes contribute to an immense metabolic burden and the need for functional mitochondria, ROS′ major primary production site. Besides that, the accumulation of α-synuclein itself provokes the generation of mtROS. Data of transgenic mouse models could prove that α-synuclein aggregation diminishes complex I activity and increase ROS levels (Hsu et al., 2000; Martin et al., 2006) before dopaminergic neuron loss (Subramaniam et al., 2014). Inhibition of complex I, ROS production, and neuronal cell death *via* apoptosis and autophagy was also linked to the presence of PD-associated gene mutations [α-synuclein (SNCA), LRRK2, PARK7, PARK2, PINK1] (Müftüoglu et al., 2004; Yamada et al., 2004; Iaccarino et al., 2007; Hayashi et al., 2009; Ho et al., 2009; Gusdon et al., 2012; Venderova and Park, 2012; Dias et al., 2013; Blesa et al., 2015; Gegg and Schapira, 2016). Oxidative stress by complex I inhibition can be pharmacologically mimicked by environmental toxins, including 1-methyl-4-phenyl-1,2,3,6-tetrahydropyridin (MPTP) and rotenone (Mizuno et al., 1987; Schapira et al., 1990; Ramsay et al., 1991; Richardson et al., 2005), leading to superoxide generation and reduction of ATP synthesis. Treatment with these substances shows similar upregulation of cell death pathways as seen in PD (Hayley et al., 2004; Clayton et al., 2005; Perier et al., 2005).

### 3.4 Cancer

#### 3.4.1 Clinical Significance

While malignant tumors can occur at any age, cancer disproportionately strikes the elderly aged 65 years or older. The median age of cancer-related death is 71–77 years, independent of sex and race (Yancik, 2005). Consequently, in an aging society, the cancer burden is still expected to increase in the following centuries. The hallmarks of cancer include genome instability, mutations, replicative immortality, and cell death resistance, as well as angiogenesis, deregulation of cellular metabolism, tumor-promoting inflammation, and avoidance of immune destruction. Therefore, cancer cells exhibit high rates of proliferation, invasion, and metastasis (Somarelli et al., 2020). Mitochondria provide energy and building blocks for new cells and modulate ROS homeostasis, oncogenic signaling, and apoptosis. Consequently, these organelles are essential in cancer development and progression (Zong et al., 2016). While the Warburg effect suggested that cancer is accompanied by mitochondrial defects, forcing cancer cells into increased aerobic glycolysis, it was proven in the following years that cancer cells still exhibit functional mitochondria and also rely on mitochondrial respiration to obtain sufficient energy (Mazurek, 2011). Mitochondria also seem to play a crucial role in the development of tumors by triggering cell integrity loss through mutations in mtDNA and the generation of ROS (Badrinath and Yoo, 2018). In line with these *in vitro* findings, clinical trials revealed that oxidative stress correlates with the development and progression of various cancer types, including colorectal cancer (Boakye et al., 2020; Janion et al., 2020), bladder cancer (Wigner et al., 2021), breast cancer (Lee et al., 2017), and prostate cancer (Oh et al., 2016).

#### 3.4.2 Cellular Mechanisms and Signaling

Cancer cells exhibit significantly higher levels of ROS than corresponding non-cancerous cells. This increase in ROS is triggered by enhanced metabolic rate, gene mutation, and hypoxia (Perillo et al., 2020). Adaption to these excessive ROS conditions is achieved by cancer cells through enhanced antioxidant capacity. Therefore, the main transcription factor involved in the antioxidant defense, the nuclear factor erythroid 2-related factor 2 (NRF2), is often upregulated in cancer cells and helps *via* boosting antioxidant defense mechanisms the proliferation of cancer cells (Jaramillo and Zhang, 2013). Besides, glutathione and thioredoxin antioxidant pathways synergize to drive cancer initiation and progression. For instance, inhibition of glutathione synthesis resulted in enhanced levels of thioredoxins. Consequently, just combined blockage of glutathione and thioredoxins resulted in cancer cell death *in vitro* and *in vivo* (Harris et al., 2015). Besides, the antioxidant potential might be further promoted through NADPH production. Interestingly, pyruvate kinase M2 was found to be predominantly present in a dimeric state in cancer cells, exhibiting less activity and forming a bottleneck in glycolysis. Consequently, the glucose flux gets redirected to the pentose phosphate pathway, where NADPH is produced (Mazurek et al., 2005; Hawk et al., 2016). Thereby, the antioxidant potential of cancer cells gets again improved.

### 3.5 Inflammation and Pain

#### 3.5.1 Clinical Significance

Inflammation is the body’s response to pathogens, external injuries, damaged cells, or irritants. Thereby, immune cells release various inflammatory mediators to dilate small blood vessels. The thereby increased blood flow allows the transport of immune system cells to the affected tissues for repair (Abdulkhaleq et al., 2018). The release of proinflammatory and immune-active substances like cytokines and chemokines can result in nerve irritation and pain signals (Abdulkhaleq et al., 2018). For instance, the proinflammatory mediator histamine is well-known to induce itching (Shim and Oh, 2008). While injuries or infections might cause intermittent increases in inflammation, chronic systemic inflammation represents the main cause for diseases such as CVD, diabetes mellitus, cancer, chronic kidney and liver diseases, and autoimmune and neurodegenerative disorders, which strongly contribute to disability and mortality worldwide (Furman et al., 2019). Inflammatory processes induce oxidative stress and reduce cellular antioxidant capacity, causing loss of tissue integrity, impaired protein function, and DNA damage. Consequently, the combination of chronic inflammation and oxidative stress is a major hallmark of age-related diseases (Khansari et al., 2009). For instance, oxidative stress parameters like enhanced levels of malonaldehyde and superoxide anions and decreased SOD activity correlated with inflammation markers, including high sensitive C-reactive protein and fibrinogen, in patients with coronary heart disease (Kotur-Stevuljevic et al., 2007). Also, a correlation between inflammation and ROS levels was suggested as a universal parameter in patients with infective endocarditis (Ostrowski et al., 2012). Besides, spontaneous ROS production by neutrophils was associated with low-grade inflammation in the elderly (Ogawa et al., 2008), emphasizing that ROS production might represent a crucial aspect in age-associated immune dysregulation.

#### 3.5.2 Cellular Signaling and Mechanisms

While pro-inflammatory processes increase ROS production (Yang et al., 2007), ROS are, in turn, also involved in the activation of pro-inflammatory processes such as mast cell activation and subsequent release of pro-inflammatory mediators (Son et al., 2006). For instance, hydrogen peroxide was shown to modulate the NF-kB signaling pathway associated with an inflammatory response. Several ROS-induced modifications were found at NF-kB, leading to activation of the NF-kB at the early phase of oxidative stress but attenuation of NF-kB activity in case of sustained stress (Lingappan, 2018). In addition, ROS are also utilized by inflammatory agonists as signaling molecules. For instance, oxidative stress produced by polymorphonuclear neutrophils during inflammation causes the opening of inter-endothelial junctions and thereby allows inflammatory cells to pass the endothelial barrier. Then, the migrated inflammatory cells contribute to the clearance of pathogens and also lead to tissue injury. Besides, ROS quickly react with nitric oxide to generate reactive nitrogen species, which induces nitrosative stress, and so adds to the pro-inflammatory impact of ROS (Mittal et al., 2014). Moreover, mitochondrial ROS production was found to convey lipopolysaccharide-driven production of proinflammatory cytokines (Bulua et al., 2011) and was identified as a major step in the activation of inflammasomes, multiprotein oligomers, which promote the secretion of pro-inflammatory cytokines (Yang et al., 2019). Acute inflammations are often induced by infections caused by bacteria, viruses, protozoa, or fungi. Thereby, ROS are used by the immune system to protect the host organism against infections. In response to inflammatory processes, phagocytes reside within the tissue to phagocyte microbes. Dependent on the microbe, phagocytes generate ROS signals to directly kill the microbes and activate ROS bursts within the cell to induce respective signaling cascades in order to eliminate pathogenes by non-oxidative mechanisms (Paiva and Bozza, 2014). Besides, ROS and peroxynitrite are also directly engaged in the nociceptive signaling by altering protein kinase A and calcium/calmodulin-dependent protein kinase type II-mediated signaling, glutamatergic neurotransmission, transient receptor potential cation channel subfamily V member 1 (TRPVI) sensitization, and cyclooxygenase enzyme (COX) activation (Salvemini et al., 2011). Interestingly, clinical trials revealed that administration of SOD1, known as the drug orgotein, reduced symptoms of osteoarthrosis of the knee joint significantly better than methylprednisolone acetate (Gammer and Brobäck, 1984), emphasizing the crucial role of ROS in the development of pain and the potential of ROS manipulation as a treatment of pain.

## 4 Reactive Oxygen Species Modulation by Approved Drugs

### 4.1 Cardioprotective Drugs

#### 4.1.1 Beta-Blocker

Beta-blockers comprise compounds that inhibit the activation of β-adrenergic receptors by endogenous catecholamines. The first beta blocker, propranolol, was approved in the 1960s to treat angina pectoris and revolutionized the treatment of CVD. Nonselective β1 and β2 adrenoreceptor blockers like propranolol and carvedilol and specific β1 adrenoreceptor blockers such as atenolol, metoprolol, and bisoprolol are nowadays widely used to ameliorate cardiac function and reduce the mortality rate in heart failure patients (Srinivasan, 2019). Moreover, beta-blockers are widely used medications to treat hypertension. Stimulation of β1 receptors induces positive chronotropic and inotropic effects in the heart muscle and modulates arterial vasoconstriction by the release of renin in the kidney. β2-adrenergic receptors are located in various organs, including the liver and vascular smooth muscle, and β2 receptor activation causes smooth muscle relaxation (Farzam and January 2021). Beta-blockers affect ROS homeostasis indirectly *via* different mechanisms. *First*, inhibition of β1 adrenergic receptors prevents oxidative stress due to catecholamine-induced reactions. Notably, ROS might be essential in conveying the action of catecholamines by adjusting the homeostasis of mitochondrial iron, critical for rate-limiting enzymes of the TCA cycle and for the mitochondrial electron transport chain (Tapryal et al., 2015). However, elevated levels of the catecholamines adrenaline and noradrenaline are associated with enhanced oxidative stress and were found in various cardiovascular dysfunctions and diseases, including tachycardia, arrhythmias, heart failure, and ischemic reperfusion injury (Nakamura et al., 2011). For instance, incubation of freshly isolated rat cardiomyocytes with adrenaline boosted the activity of mitochondrial complexes and caused increased expression of SOD2 after 3 h of incubation, potentially due to enhanced electron leakage from the ETC and a boost in ROS production (Costa et al., 2009). *Second*, beta-blocker might reduce ROS production indirectly by lowering mechanical stress in vessels. Cyclic stretching increased ROS and a ROS-dependent activation of a signaling cascade, including extracellular signal-regulated kinases (ERK1/2) and JNK in neonatal rat ventricular myocytes (Pimentel et al., 2001). *Third*, the nonselective β1 and β2 adrenoreceptor blocker carvedilol was found to scavenge ROS directly and might also inhibit α1 stimulated hypertrophic signaling mediated by ROS (Nakamura et al., 2011). Due to its pleiotropic effects, including antioxidant actions or enhancement of insulin sensitivity (Nguyen et al., 2019), carvedilol was speculated to be more effective than other beta-blockers like metoprolol or bisoprolol in reducing the mortality rate in humans (Rain and Rada, 2015). However, a clinical trial in patients with chronic systolic heart failure revealed carvedilol to be less effective than bisoprolol in decreasing levels of troponin T, ameliorating inflammation, and increasing forced expiratory volume. Nevertheless, the impact of carvedilol on oxidative stress markers was more pronounced (Toyoda et al., 2020). For instance, carvedilol was applied in patients with dilated cardiomyopathy and enhanced oxidative DNA damage, significantly reducing oxidative DNA damage, lipid peroxidation and ameliorating heart failure (Nakamura et al., 2002; Kono et al., 2006). Besides carvedilol, also the β1-selective beta-blocker nebivolol was shown as a direct antioxidant either by scavenging free radicals or by acting as a chain breaker through proton donation or electron stabilization (Gao and Vanhoutte, 2012). Besides, nebivolol was found to inhibit ROS formation by reducing the activity and expression of the vascular NOX in angiotensin II-treated animals and cells (Oelze et al., 2006). Notably, the ratio of reduced glutathione to oxidized glutathione was significantly increased in patients with essential hypertension after treatment with carvedilol, while nebivolol-treated patients did not show significant differences in this parameter but showed increased nitrogen dioxide plasma concentrations (Zepeda et al., 2012). These reports suggest that the proper use of different beta-blockers might be dependent on the individual pathophysiology.

#### 4.1.2 ACE Inhibitors/AT1 Antagonists

Angiotensin-converting enzyme (ACE) inhibitors prevent the conversion of angiotensin I into angiotensin II that binds to the angiotensin II receptor (AT1) in blood vessels to mediate its vasoconstrictive effect (Burnier, 2001), whereas AT1 receptor antagonists directly inhibit the binding of angiotensin II to AT1 receptors (Gradman, 2002). Consequently, ACE inhibitors are used to control blood pressure to reduce mortality in patients with congestive heart failure and in patients with high cardiovascular risk profiles, including diabetes. Moreover, ACE inhibitors are essential in delaying the progression of chronic renal diseases since they lower proteinuria. The first orally active ACE inhibitor, captopril, got approved by the FDA in 1981. Similar effects as for ACE inhibitors could be achieved by applying AT1 receptor antagonists, first approved by the FDA as losartan in 1995 (Ripley and Hirsch, 2010). ACE inhibitors and AT1 antagonists are supposed to diminish the angiotensin II-mediated generation of ROS and partly also directly scavenge ROS production. Angiotensin II was found to modulate the pressor effect through ROS signaling in the glutamatergic neuron in stress-induced hypertensive rats. Thereby, NAPDH oxidase-derived ROS activates the SAPK and the JNK, promoting the expression of AT1 receptors in glutamatergic neurons. Consequently, glutamate gets released into the spinal cord and leads to the pressor response (Jiang et al., 2018). Besides, the AT1 receptor antagonist candesartan was found to blunt the tumor necrose factor α (TNFα)-induced inflammatory cytokine production of embryonic kidney epithelial cells by inhibiting oxidative stress. Notably, knockdown of the AT1 receptor did not alter candesartan’s impact on ROS activity in humans (Yu et al., 2019). Angiotensin II was found to enhance ROS formation *via* AT1 receptor activation in old sheep, which was counteracted by the application of ACEII inhibitors (Gwathmey et al., 2010). Moreover, disruption of the AT1 receptor in mice caused reduced oxidative damage and significantly promoted longevity (Benigni et al., 2009). Application of the ACE inhibitor lisinopril attenuated ROS formation and counteracted cardiovascular remodeling in diabetic rats to the same extent as the antioxidant N-acetyl-l-cysteine (NAC) (Fiordaliso et al., 2006). Notably, combined application of the ACE inhibitor temocapril with the AT1 antagonist olmesartan induced a more pronounced suppression of ventricular hypertrophy and fibrosis in a diastolic heart failure rat model in comparison to the monotherapy with temocapril. This benefit was associated with an additive effect on the blockage of ROS generation and inflammation signaling (Yoshida et al., 2004). Besides, it was discussed whether thiol-carrying compounds like alacepril might function as direct ROS scavenging agents. For instance, 0.6–0.7 mM of alacepril reduced ROS production in bronchoalveolar lavage cells from chronic obstructive pulmonary disease patients by 50%, while 3–4 mM of thiol-free lisinopril was necessary to achieve the same effect (Teramoto et al., 2000), suggesting that Moreover, thiol-carrying captopril was more effective against copper-induced oxidative modification on lipids and proteins than the non-thiol ACE inhibitors enalapril and lisinopril (Fernandes et al., 1996). However, the non-thiol carrying AT1 receptor antagonist candesartan inhibited oxidative stress in embryonic kidney epithelial cells independent of AT1 receptor activity (Yu et al., 2019). Consequently, it remains questionable whether the thiol-group is necessary for the direct antioxidant properties of some ACE inhibitors and AT1 antagonists.

#### 4.1.3 Statins

Statins inhibit the rate-limiting step in cholesterol synthesis by blocking the liver enzyme 3-hydroxy-3-methyl-glutaryl-coenzyme A (HMG-CoA) reductase that converts HMG-CoA to mevalonic acid. Consequently, cholesterol levels in the blood drop, and the potential side effects of hyperlipidemia are prevented. The first statin approved by the FDA as therapy for preventing coronary atherosclerotic events was lovastatin in 1987 (Harrington, 2017). HMG-CoA reductase is a crucial enzyme in the mevalonate pathway. Blocking of HMG-CoA reductase also reduces the bioavailability of various products of the mevalonate pathway, including heme A, ubiquinone, isoprenoids, corticosteroids, and vitamin D. Consequently, statins affect mitochondrial function indirectly *via* mevalonate pathway metabolite depletion. Besides, also direct impairment of the ETC activity was reported (Mollazadeh et al., 2021). Notably, statins exhibit opposite effects on mitochondria of cardiac and skeletal muscles (Sirvent et al., 2005; Bouitbir et al., 2012). A different effect on mitochondrial respiration is discussed as a potential reason. For instance, simvastatin was found to inhibit complex I of the ETC in human and rat skeletal muscle samples, while cardiomyocytes remained largely unaffected (Sirvent et al., 2005). Notably, statins were found to trigger a *mitohormetic* response by a transient ROS signaling in cardiac tissues, resulting in the upregulation of ROS detoxifying enzymes. At the same time, an enhanced level of ROS displayed harmful actions on skeletal muscle tissues (Bouitbir et al., 2012). In line with this finding, ROS production was decreased and oxidative capacities and peroxisome proliferator-activated receptor-gamma activator 1 (PGC-1) expression enhanced in the atrium of patients treated with atorvastatin, while statin-induced muscular myopathy was accompanied by reduced oxidative capacities, a side-effect counteracted by the application of antioxidant molecules (Bouitbir et al., 2012). Analysis of skeletal muscle biopsy samples from patients with statin-associated myopathy confirmed an enhanced hydrogen peroxide production after statin treatment. However, a difference between slow- and fast-twitching muscle fibers fueled by a greater extent of glycolysis could be detected: Atorvastatin treatment increased hydrogen peroxide accumulation, decreased GSH/GSSG ratios, and triggered apoptotic pathways in the glycolytic plantaris muscle of rats, while the oxidative soleus muscle was largely unaffected due to high antioxidative capacity (Bouitbir et al., 2016). Consequently, the response of different cell types to statin-induced initial ROS burst is probably dependent on the individual metabolic state and potential for antioxidant actions. Thereby, oxidative muscle fibers might be better adjusted to enhanced mitochondrial activity and to related side products like ROS. For instance, an increase in ROS is limited in cardiac myofibres due to their highly efficient antioxidant systems, causing PGC-1α activation and, thereby, mitochondrial biogenesis and function, while cells with lower antioxidant capacity face a giant ROS burst and cellular damage (Mollazadeh et al., 2021). Treatment with antioxidants might be a potential solution to counteract the destructive action of statins in muscle fibers. However, a clinical study revealed that tocopherol supplementation in addition to pravastatin treatment for half a year did not further improve lipid levels or the frequency of adverse effects, including muscle damage in older adults (Carlsson et al., 2002), highlighting that boosting cellular antioxidant defense mechanisms might be a more promising strategy to counteract potential side-effects of statins. Besides, lipophilic statins like cerivastatin, fluvastatin, atorvastatin, and simvastatin specifically decreased glutamate-driven state 3 respiration and induced mitochondrial swelling, cytochrome c release, and DNA fragmentation in rat skeletal muscle cells. In contrast, the hydrophilic pravastatin did not impair mitochondrial function (Kaufmann et al., 2006). Based on these results, administration of hydrophilic statins might prevent mitochondrial accumulation and, thus, harmful effects on the skeletal muscles.

#### 4.1.4 Platelet Aggregation Inhibitors (“Antiplatelets”)

Antiplatelet medications are used in the primary and secondary prevention of thrombotic diseases by decreasing platelet aggregation and thrombus formation. Oral antiplatelets are classified regarding their mechanism of action, including platelet aggregation inhibitors aspirin and clopidogrel. Low-dose aspirin, 75–150 mg once a day (Cryer, 2002), is the most prominent antiplatelet drug, suppressing prostaglandin and thromboxane production through irreversible inactivation of COX. Thereby, the formation of the pro-thrombotic thromboxane A2 in platelets is blocked (Iqbal et al., 2021). Approved around 1900 to treat fever, pain, and inflammation, the compound experienced a revival in the last decades of the 20th century by widespread use as prevention against cardiovascular incidents (Tsoucalas et al., 2011). Nowadays, low-dose aspirin is one of the most popular antiplatelet therapies for the treatment of patients with the acute coronary syndrome (Amsterdam et al., 2014; Roffi et al., 20152016) and for the prevention of atherothrombotic complications in high-risk patients (Parekh et al., 2013; Patrono, 2015). Aspirin was found to inhibit ROS production by downregulation of NOX4 and the inducible nitric oxide synthase in human endothelial cells exposed to oxidized LDL (Chen et al., 2012). Besides, low doses of aspirin also increased the expression of SOD1 and SOD2 in rat astrocytes treated with the toxic peptide Aβ (Jorda et al., 2020). Notably, a double-blind, randomized study revealed that the combined application of low-dose aspirin with vitamin E caused a significant reduction in platelet adhesiveness compared to aspirin only. However, the incidence of hemorrhagic strokes increased in patients treated with both drugs, reaching no statistical significance due to the low number of cases (Steiner et al., 1995). These findings highlight the potential of antioxidant defense mechanisms in the prevention of platelet aggregation but once again question the usage of ROS scavengers.

### 4.2 Oral Antidiabetic Drugs

#### 4.2.1 Metformin

Metformin was first approved in the United Kingdom in 1958 and got the first-line therapy for T2DM (Scarpello and Howlett, 2008). Metformin shows versatile anti-diabetic effects in various organs and tissues (Foretz et al., 2019). Firstly, metformin impairs glucose absorption in the intestine by increasing the secretion of glucose-lowering hormone glucagon-like peptide (GLP-1) (Mannucci et al., 2004). Secondly, metformin promotes glucose uptake in peripheral tissues by glucose transporter 4 (GLUT4) translocation to the plasma membrane, which allows increased blood glucose clearance and thus counteracts glycemia (Musi et al., 2002; Natali and Ferrannini, 2006; Boyle et al., 2011; Grzybowska et al., 2011; Lee et al., 2012b; Mummidi et al., 2016). Consequently, metformin attenuates fasting plasma insulin levels and helps to restore insulin sensitivity (Grzybowska et al., 2011). Thirdly, metformin inhibits complex I of the respiratory chain, causing an energy deficit leading to the activation of AMP-activated protein kinase (AMPK) (El-Mir et al., 2000; Owen et al., 2000; Zhou et al., 2001). AMPK signaling, in turn, hampers glucose production by inhibition of mitochondrial glycerol-3 phosphate dehydrogenase (Madiraju et al., 2014) and glucagon-stimulated hepatic gluconeogenesis (Miller et al., 2013). Besides that, pAMPK signaling regulates multiple other downstream pathways such as (Jaul and Barron, 2017) nutrient sensing by inhibition of mTORC1 and activation of SIRT1, as well as (Hayyan et al., 2016) initiation of mitochondrial biogenesis through PGC1*α*, (Tejero et al., 2019), inhibition of pro-inflammatory signaling *via* NFkB, and (Sun et al., 2010) the regulation of autophagy (Semancik and Vanderwoude, 1976; Suwa et al., 2006; Salminen and Kaarniranta, 2012; Aatsinki et al., 2014; Barzilai et al., 2016; Zhou et al., 2016; Herzig and Shaw, 2018). In addition, metformin-induced AMPK signaling stabilizes the transcription factor NRF2 (Onken and Driscoll, 2010), a master regulator of redox regulations, consequently initiating the expression of antioxidant genes such as CAT, GSH, SOD (Ashabi et al., 2015). The upregulation of antioxidant defense enzymes eventually leads to a decrease in ROS, an impairment of NOX, and a boost of SOD expression (Diniz Vilela et al., 2016; Shin et al., 2017). Inhibition of NOX by metformin highly contributes to the regulation of redox homeostasis as NOX-derived ROS represents the primary source of high glucose-induced oxidative stress (Inoguchi et al., 2000; Kim et al., 2002; Inoguchi et al., 2003). In addition, metformin interferes with RNS production and thereby diminishes nitro-oxidative stress. For instance, it was shown that the bioavailability of nitric oxide, a contributor to endothelial function, was improved by metformin, while levels of cytotoxic peroxynitrite were decreased in diabetic rats (Sambe et al., 2018).

In accordance with metformin-dependent activation of AMPK signaling and the consequent induction of redox regulatory processes, several *in vitro* studies presented antioxidant effects of metformin (Marycz et al., 2016; Ahangarpour et al., 2017; Smieszek et al., 2017; Algire et al., 2012; Abd-Elsameea et al., 2014). A randomized clinical trial investigating the impact of metformin on ROS homeostasis of T2DM patients found an improvement in the antioxidant status and a cardioprotective effect upon metformin treatment (Chakraborty et al., 2011). In addition to the interference with redox regulatory processes, metformin itself displays direct antioxidant actions by detoxifying hydroxyl radicals, as seen in murine *in vitro* and *in vivo* models of oxidative liver injury and cardiac fibrosis and human monocytes/macrophages (Mummidi et al., 2016; Dai et al., 2014; Buldak et al., 2014). Metformin supplementation was further linked to anti-inflammatory and anti-apoptotic processes in several studies investigating neurodegeneration and multiple sclerosis (Nath et al., 2009; Ullah et al., 2012; Alzoubi et al., 2014). Metformin interferes with the production of IL1β, a pro-inflammatory cytokine responsible for pancreatic β-cell apoptosis (Kelly et al., 2015). This mechanism counteracts ROS-dependent increases in IL1β expression in an AMPK-independent fashion (Bauernfeind et al., 2011). The same study revealed that metformin raises levels of the anti-inflammatory cytokine IL-10 (Bauernfeind et al., 2011). Clinical studies observed metformin’s role in preserving cognitive function (Ng et al., 2014), resulting in reduced depressive behavior (Guo et al., 2014) and decreased mortality in diabetic patients (Barzilai et al., 2016). Importantly, metformin-dependent health benefits go beyond glycemic control and include beneficial effects against various types of cancer (Heckman-Stoddard et al., 2017), CVD (Rena and Lang, 2018), neurodegenerative disorders (Rotermund et al., 2018), and autoimmune diseases (Ursini et al., 2018). Several preclinical and clinical studies have strong evidence for a geroprotective potential of metformin (Barzilai et al., 2016; Glossmann and Lutz, 2019; Piskovatska et al., 2019; Soukas et al., 2019). In addition, epidemiological and association studies show that metformin is linked to reduced incidences and all-cause mortalities in several age-related diseases, such as age-associated cancers and AD (Barzilai et al., 2016; Campbell et al., 2017; Valencia et al., 2017). Based on these promising results, the “targeting aging with metformin” (TAME, ClinicalTrials.gov Identifier: NCT02118727) study currently investigates metformin’s potential in aging and its therapeutical potential in age-related diseases (Campisi et al., 2019; Wang et al., 2020).

#### 4.2.2 DPP4 Inhibitors and GLP-1 Agonists

Inhibitors of dipeptidyl peptidase 4 (DPP4), so-called gliptins, and glucagon-like protein 1 (GLP-1) receptor agonists (GLP-1RAs), also known as incretin mimetics, represent two drug classes that tackle the same pathway by acting in opposing ways. While gliptins increase the stability of GLP-1 by preventing its degradation, GLP-1RAs mimic GLP-1 and thus promotes glucagon suppression and insulin secretion (Deacon et al., 2012). DPP4 is a serine protease responsible for the degradation of proteins, including the incretins GLP-1 and gastric inhibitory peptide (GIP), two metabolic hormones involved in the attenuation of blood glucose levels. Elevated DPP4 activity is a risk factor for developing metabolic syndrome and T2DM (Zheng et al., 2014) and is associated with insulin resistance (Sell et al., 2013). As a result, DPP4 deficiency in mice manifests in improved glucose tolerance (Marguet et al., 2000) and decreased obesity and insulin resistance (Conarello et al., 2003). Besides lowering plasma insulin levels, treatment with the DPP4 inhibitors vildagliptin and sitagliptin successfully improved oxidative stress parameters in obese insulin-resistant rats (Apaijai et al., 2013). Another study reported that vildagliptin and sitagliptin positively affected mitochondrial oxidative stress and mitochondrial function, resulting in enhanced cognition and hippocampal brain function in high-fat diet-induced insulin-resistant Wistar rats (Pintana et al., 2013). Advanced glycation endproducts (AGEs) represent a measure of oxidative stress in T2DM. It was shown that crosstalk between AGEs, the receptor for AGEs (RAGE), and the DPP4-incretin system adds up to diabetic vascular complications (Yamagishi et al., 2015). Thereby, DPP4 positively correlates with ROS production and RAGE gene expression (Ishibashi et al., 2013). This process could be reversed by DPP4 inhibition *via* linagliptin supplementation in endothelial cells (Ishibashi et al., 2013). Similar results were obtained upon teneligliptin treatment resulting in reduced adverse effects of AGEs in mouse peritoneal macrophages and THP-1 cells (Terasaki et al., 2020). In general, oxidative stress markers and inflammatory cytokines were attenuated in T2DM patients receiving DPP4 inhibitor treatment for 4–16 weeks (Rizzo et al., 2012; Tremblay et al., 2014). Besides counteracting oxidative stress, DPP4 inhibitors improve mitochondrial function in rats on a high-fat diet (Apaijai et al., 2013; Pintana et al., 2013; Pipatpiboon et al., 2013) and increase mitochondrial biogenesis and exercise capacity in a mouse model for ischemic heart failure (Takada et al., 2016). Similar to this, GLP-1 agonists stimulated mitochondrial biogenesis and antioxidant defense systems by modulation of PPAR signaling in PC12 cells and mice treated with GLP-1RA (An et al., 2015). This manifests in increased mitochondrial mass and function associated with improved pancreatic β-cell function in INS-1 rat insulinoma cells (Kang et al., 2015). From a mechanistic point of view, treatment with the GLP-1 agonist extendin-4 resulted in upregulation of superoxide dismutase and protected against ROS-induced apoptosis in adipose-derived mesenchymal stem cells (Zhou et al., 2014). Moreover, the GLP-1 agonists, liraglutide, D-ser2-oxyntomodulin, a GLP-1/GIP dual receptor agonist, dAla (2)-GIP-GluPal, Val(8)GLP-1-GluPal and exendin-4 enhanced the expression of the autophagy-associated marker protein atg7 and pyruvate dehydrogenase and improved mitochondrial function in neuronal SH-SY5Y cells (Jalewa et al., 2016).

#### 4.2.3 Glitazones

Glitazones, also known as thiazolidinediones (TZDs), are approved antidiabetic drugs and include compounds such as rosiglitazone and pioglitazone (Hauner, 2002). They represent specific agonists of the peroxisome proliferator-activated receptor *γ* (PPARγ) and thereby modulate its downstream metabolic regulations (Day, 1999). Their hypoglycemic and antidiabetic effects result from increased glucose absorption and insulin sensitivity in peripheral tissues (Hauner, 2002). Similar to metformin, TZDs were described to inhibit complex I of the respiratory chain in *vitro* activity assays (Brunmair et al., 2004) and promote cell survival by maintaining the *Ψ*_mito_ *via* PPARγ signaling in lymphocytes (Wang et al., 2002). Pioglitazone was shown to counteract oxidative stress and inflammation, increase mitochondrial biogenesis in non-alcoholic fatty liver disease (Bogacka et al., 2005), and attenuate mitochondrial-induced oxidative damage in human subcutaneous adipose tissue human neuron-like cells (Bogacka et al., 2005; Ghosh et al., 2007). In accordance with these findings, pioglitazone increases the SOD1 activity and inhibits NOX expression in rat mesangial cells (Wang et al., 2013). Bolten *et al.* (Bolten et al., 2007) concluded that observed hypoglycemic effects are more likely a consequence of improved mitochondrial function rather than PPAR*γ* signaling. In contrast, treatment of human hepatoma cells with troglitazone caused severe side effects and mitochondrial structure injuries, which were less potent upon treatment with rosiglitazone or pioglitazone in similar concentrations (Hu et al., 2015). As a consequence, troglitazone was withdrawn as an antidiabetic drug due to hepatotoxicity and mitochondrial toxicity side effects just 3 years after its approval in 2000 (Hu et al., 2015).

#### 4.2.4 SGLT2 Inhibitors

Glucose is re-absorbed *via* active or passive transport processes during blood filtration in the proximal renal tubule of kidneys (Vallon and Thomson, 2017). One crucial player during this process is the sodium-glucose cotransporter 2 (SGLT2) (Kalra, 2014), which can be pharmacologically inhibited by SGLT2 inhibitors. Such compounds prevent the re-uptake of glucose and favor glucose secretion independent of insulin (Chao, 2014), thus representing antidiabetic drugs to counteract glycemia. Furthermore, SGLT2 inhibitors impair gluconeogenesis and increase insulin sensitivity and insulin secretion of β-cells (Han et al., 2008; Ferrannini et al., 2014; Wilding et al., 2014; Kern et al., 2016). More importantly, SGLT2 inhibitors comprise antioxidant properties by reducing free radical production and strengthening the antioxidant system (Osorio et al., 2012; Ishibashi et al., 2016). Experiments in mice revealed an improved redox state, diminished oxidative damage (Sugizaki et al., 2017), and enhanced mitochondrial function, eventually leading to a balanced ROS homeostasis in the brain (Sa-Nguanmoo et al., 2017). Mechanistically, SGLT2 inhibitors affect the activity and expression of prooxidant enzymes such as NOX, eNOS, and XO (Oelze et al., 2014; Kawanami et al., 2017). For instance, empagliflozin treatment in diabetic rat models led to the downregulation of NOX1 and NOX2 (Oelze et al., 2014). Moreover, NOX4 expression was shown to be impaired by dapagliflozin (Steven et al., 2017). In both cases, free-radical generation and oxidative damage are counteracted (Habibi et al., 2017; Steven et al., 2017). In addition to the depletion of prooxidant processes, SGLT2 inhibitors also strengthen the antioxidant defense system. Several studies show that expression of CAT, SOD, and GPX are increased in diabetic animal models in the presence of phlorizin (Osorio et al., 2012), dapagliflozin (Shin et al., 2016), and TA-1887 (Sugizaki et al., 2017), another SGLT2 inhibitor.

#### 4.2.5 Alpha-Glucosidase Inhibitors

Alpha-glucosidase inhibitors, such as acarbose and miglitol, delay the digestion of carbohydrates by inhibiting alpha-glucosidase enzymes in the small intestines and thereby preventing postprandial hyperglycemia. The alpha-glucosidase inhibitor acarbose reduces inflammatory cytokine production, as seen in reduced levels of interferon-gamma induced protein 10 kD, monocyte chemoattractant protein-1, macrophage-derived chemokines, TNFα as well as NF-kB activity in THP-1 cells (Lin et al., 2019). Moreover, it was observed that acarbose co-treatment with insulin reduced inflammation and oxidative stress in diabetic individuals (Li et al., 2016). Reduced levels of superoxide might be the consequence of acarbose-dependent inhibition of NOXes in the aorta, heart, and kidney of obese diabetic rats (Rösen and Osmers, 2006). Furthermore, inhibition of NOX4 oxidase-dependent superoxide production was seen in rat aortic endothelial cells and is linked to anti-inflammatory regulations (Li et al., 2019).

#### 4.2.6 Sulfonylurea and Glinide

Sulfonylurea inhibits ATP-sensitive K^+^ channels in the plasma membrane of β-cells and initiates insulin release and hypoglycemia (Groop, 1992). Sulfonylureas, including gliclazide, glibenclamide, and glimepiride, do also affect ATP-sensitive K^+^ channels in the inner mitochondrial membrane and thereby modify mitochondrial function (Inoue et al., 1991; Suzuki et al., 1997; Szewczyk et al., 1997; Argaud et al., 2009). Moreover, gliclazide treatment in rat models reduces oxidative stress and inflammation *via* several mechanisms, including upregulation of antioxidant enzymes such as SOD, CAT, and GPX1 (Del Guerra et al., 2007; Alp et al., 2012; Araújo et al., 2019).

### 4.3 Anti-degenerative Drugs

#### 4.3.1l-Dopa (or Levodopa) and Dopamine Agonists

Until now, l-dopa is considered as “gold standard” for PD therapy (Nagatsu and Sawada, 2009). In the late 1960s, high-dose l-dopa treatment was shown to result in remarkable clinical efficacy in PD patients by restoring dopamine levels in the brain (Barbeau et al., 1961) and was first approved in 1970 (Abbott, 2010). Despite its effectiveness, long-term treatment with l-dopa often results in motor complications, including abnormal involuntary movements (Pahwa et al., 2006; Fabbrini et al., 2007). Similar to this, treatment with dopamine agonists is associated with a range of side effects, from mild to severe implications (Faulkner, 2014). It was shown that degradation of dopamine after l-dopa supplementation results in a dose-dependent increase in ROS and cell death of serotonergic neurons (Stansley and Yamamoto, 2013). These observations underline that both l-dopa and dopamine agonists are thought to act symptomatically only (Bonuccelli, 2003; Segawa et al., 2003; Nagatsu and Sawada, 2009; Blandini and Armentero, 2014), emphasizing the urgent need for more potent drugs that target early dysregulations of the disease, such as oxidative stress.

#### 4.3.2 MAO-B Inhibitors

MAO-B-inhibitors against PD include the irreversible inhibitor selegiline (L-deprenyl), which was first approved by the FDA in 1996, followed by rasagiline in 2006 (Knudsen Gerber, 2011), as well as safinamide, the first reversible FDA-approved MAO-B inhibitor against PD available for clinical use since 2015 (Deeks, 2015). In preclinical models, selegiline was shown to increase levels of antioxidant enzymes such as glutathione and SOD, improve oxidative stress biomarkers, and reduce neuronal loss in rats (Kumar et al., 2018; Ahmari et al., 2020). Similar effects were obtained with rasagiline which attenuated oxidative stress in rats, as measured by levels of 7-ketocholesterol and GSSG/GSH ratio (Aluf et al., 2013).

Clinical trials with safinamide alone or in combination with levodopa or dopamine agonists (pergolide, ropinirole, pramipexole, cabergoline) confirmed improved PD symptoms (Martínez-Martín et al., 1994; Stocchi et al., 2006; Wasan et al., 2021). However, the clinical potential of MAO-B inhibitors to attenuate oxidative stress by inhibiting MAO-induced hydrogen peroxide production remains to be shown as clinical evidence of improved oxidant status in PD patients is lacking. Consequently, it is questionable whether observed positive effects with MAO-B inhibitors are due to neuroprotection or instead limited to symptomatic benefits such as maintaining dopamine levels (Schulzer et al., 1992; Shoulson, 1992; Stocchi et al., 2006).

#### 4.3.3 Repurposing of Antidiabetics as Antidementia Drug

Notably, AD and PD show alterations in oxidative stress levels, hyperglycemia, mitochondrial dysfunction, glucose metabolism, insulin signaling, insulin resistance, and inflammatory processes (Baker et al., 2011; Moran et al., 2013; Willette et al., 2015; Morsi et al., 2018; Sergi et al., 2019; Cheng et al., 2020). Brains of AD individuals show a deficiency of GLUT1 and GLUT3 expression (Simpson et al., 1994) as a result of decreased activity of enzymes involved in glycolysis and TCA cycle (Manczak et al., 2004; Bubber et al., 2005; Manczak and Reddy, 2012). Moreover, AD brains often exhibit impaired insulin receptors activity (Frölich et al., 1998; Talbot et al., 2012), attenuated levels of insulin and insulin growth factor 1 as well as decreased levels of downstream proteins such as insulin receptor substrate 1 (Rivera et al., 2005; Moloney et al., 2010; Talbot et al., 2012). These characteristics positively correlate with cognitive impairments (Talbot et al., 2012) and the progression of AD (Rivera et al., 2005). In conclusion, T2DM and AD share common derangements in glucose metabolism, which led to the term “type III diabetes” and the classification of AD as a metabolic disease that might alternatively be treated with antidiabetics as a novel therapeutic strategy (de la Monte and Wands, 2005). Similar to AD, PD has overlapping dysregulations with diabetes. For example, 50–80% of PD patients have decreased glucose tolerance (Sandyk, 1993) and impaired glucose metabolism, which is considered an early event in the pathology of PD (Borghammer et al., 2012; Dunn et al., 2014). An integrative network analysis compared gene expression in PD and T2DM and elucidated dysregulation of 7 genes involved in insulin and IR signaling as a common mechanism of action (Santiago and Potashkin, 2013). Another common feature of PD and T2DM pathogenesis is mitochondrial dysfunction and impairment of the mitochondrial complex I (Esteves et al., 2008). Consequently, targeting metabolic dysregulations in neurodegenerative diseases has gained great therapeutical interest due to strong similarities to T2DM.

By strengthening the antioxidant system, metformin was shown to extinct ROS from brain tissues (Garg et al., 2017; Tang et al., 2017; Ruegsegger et al., 2019; Docrat et al., 2020) and thereby caught attention as a possible off-label treatment for AD. Indeed, preclinical animal studies suggest a role of metformin in preventing neuropathology in AD and T2DM models (Kickstein et al., 2010; Li et al., 2012; Cardoso and Moreira, 2020) as well as reducing the risk for AD development (Chin-Hsiao, 2019). Interestingly, metformin treatment interferes with amyloid plaque deposition and promotes hippocampal neurogenesis (Ou et al., 2018). In addition, it activates insulin signaling, inhibits structural changes under hyperinsulinemic conditions (Gupta et al., 2011), and restores mitochondrial function in an AMPK-dependent fashion (Chiang et al., 2016). A pilot clinical study observed that improved cognitive function, learning performance, and memory were positively correlated with metformin treatment (Koenig et al., 2017). However, these observations are controversial as other studies reported elevated Aβ levels (Chen et al., 2009) and an increased risk of AD (Imfeld et al., 2012). Thus, the effects of metformin on the pathology of AD remain to be investigated in more detail to conclude on its therapeutic potential. Metformin acts neuroprotective in the development and progression of PD (Paudel et al., 2020). These effects manifest in reduced neuroinflammation and dopaminergic cell death (Lu et al., 2016), reduced α-synuclein aggregation (Pérez-Revuelta et al., 2014; Saewanee et al., 2021), and improved cognitive and locomotor function in animals (Patil et al., 2014; Lu et al., 2016). However, clinical studies validating these metformin-specific effects in PD are still inconclusive or lacking, as metformin was most effective when combined with other antidiabetics, such as sulfonylurea (Wahlqvist et al., 2012).

### 4.4 Anti-cancer Drugs

ROS production plays an essential role in anticancer therapies. Depending on the actual level, ROS either act as tumor-suppressing or as a tumor-promoting agent (Sahoo et al., 2021). For instance, a moderate increase of intracellular ROS levels triggers cell proliferation and angiogenesis and inactivates tumor suppressor genes, promoting tumor progression (Kumari et al., 2018; Perillo et al., 2020). In contrast, overwhelming ROS levels that overcome the antioxidant defensive system of cancer cells induce cancer cell death (Galadari et al., 2017). Recent studies demonstrate that ROS levels exceeding the redox capacity might be used as anticancer therapies. Thereby, an accelerated accumulation of ROS by a selective triggering of a ROS burst or by inhibition of antioxidant processes disturbs the redox homeostasis and leads to extensive cellular damage and cell death (Wang and Yi, 2008; Trachootham et al., 2009). Enhanced ROS generation is either achieved through exogenous approaches or by endogenous ROS release (Van Loenhout et al., 2020). Several studies demonstrated that exogenous and endogenous ROS bursts actively contribute to the mechanism of action of anti-cancer therapies such as radiotherapy and chemotherapy and, therefore, enhance their efficacy (Ozben, 2007; Zhang et al., 2009; Kim et al., 2019). Physical interventions like radiotherapy or photodynamic therapy are exogenous ROS sources (Van Loenhout et al., 2020). Besides, various agents induce oxidative stress *via* endogenous ROS generation and accumulation. Compounds that generate high levels of ROS include anthracyclines including doxorubicin, platinum coordination complexes such as cisplatin, alkylating agents like cyclophosphamide, camptothecins, arsenic agents, and topoisomerase inhibitors (Weiner, 1979). Thereby, these agents either directly induce ROS generation or inhibit antioxidant defense mechanisms (Kim et al., 2019). For instance, motexafin gadolinium, doxorubicin, cisplatin, and 2-methoxyestradiol act by direct ROS generation. Thereby, motexafin gadolinium accepts electrons to form superoxide (Magda and Miller, 2006), doxorubicin induces chelation of iron to generate hydroxyl radicals (Kotamraju et al., 2002), cisplatin induces ROS generation by damaging mtDNA and the electron transport chain (Marullo et al., 2013), and 2-methoxyestradiol inhibits the ETC complex 1 (Hagen et al., 2004). Even though anticancer drugs with direct ROS-accumulating activity have been helpful in combating various cancers, their effects on normal cells remain controversial as they harm both cancer cells and normal cells (Francis et al., 2009; Cardinale et al., 2015). In contrast, the antioxidant process is, for example, inhibited by buthionine sulfoximine and imexon. Both disrupt GSH activity and disturb the *Ψ*_mito_, generating oxidative stress in cancer cells (Griffith and Meister, 1979; Moulder et al., 2010; Sheveleva et al., 2012). However, the inhibition of antioxidative enzymes also has side effects on normal cells and tissues (Dvorakova et al., 2002; Abdelhamid and El-Kadi, 2015). Consequently, it might be helpful to specifically target these agents to cancer cells *in vivo*, for instance, by using characteristic molecular signals of cancer cells. Notably, therapeutic strategies affecting ROS homeostasis are predominantly used to attack late-stage cancer cells since early-stage cancer cells are often able to actively counteract ROS disturbances by adjusting their redox status through the upregulation of antioxidant enzymes. Late-stage cancer cells already exhibit higher basal ROS levels, and additional ROS bursts are more effective to severe cytotoxic effects (Kim et al., 2019), inducing apoptosis, autophagic cell death, or necroptosis (Kim et al., 2019; Perillo et al., 2020).

### 4.5 Analgetics

Acetaminophen, also known as N-acetyl-p-aminophenol or paracetamol, is one of the most commonly used medications for pain worldwide. Approved in 2002 by the FDA, it is used as an analgesic and antipyretic agent and is recommended as first-line treatment in geriatric patients (Abdulla et al., 2013), pregnant women (Aitkin et al., 1996), and children (Cranswick and Coghlan, 2000; Purssell, 2002). Acetaminophen exerts its effects by blocking the synthesis of prostaglandins from arachidonic acid and *via* actions of its metabolite AM404 (Flower and Vane, 1972; Ghanem et al., 2016). Inhibition of prostaglandin synthesis is achieved by hampering the activity of COX-1 and COX-2 (Esh et al., 2021). COX-1 is expressed in most tissues and regulates basal levels of prostaglandins which control platelet activation and protect the lining of the gastrointestinal tract (Crofford, 1997). COX-2 is inducible and responsible for releasing prostaglandins after infection, in case of injury, or during cancer development. Prostaglandins mediate a number of biological effects, including the induction of an inflammatory immune response (Ornelas et al., 2017). In case of low levels of arachidonic acid and peroxide, therapeutic concentrations of acetaminophen inhibit COX activity sufficiently. However, acetaminophen has little effect when arachidonic acid or peroxide levels are high, as seen in severe inflammatory conditions such as rheumatoid arthritis (Boutaud et al., 2002). Accordingly, the anti-inflammatory action of acetaminophen is modest (Graham et al., 2013). Besides blocking prostaglandin synthesis, the metabolite of acetaminophen, AM404, displays analgetic effects. AMA404 is formed from 4-aminophenol by the action of fatty acid amide hydrolase and has been detected in cerebrospinal fluid of humans treated with acetaminophen (Ghanem et al., 2016; Sharma et al., 2017). AMA404 works as a weak agonist of cannabinoid receptors CB1 and CB2, as an inhibitor of endocannabinoid transporter, and a potent activator of TRPVI receptor (Anderson, 2008; Ghanem et al., 2016). Notably, acetaminophen also inhibits prostaglandin synthesis indirectly by scavenging peroxynitrite, an activator of COX (Schildknecht et al., 2008). When used in recommended doses, acetaminophen has few side effects, and iatrogenic complications are infrequent and minor (Cranswick and Coghlan, 2000; Warwick, 2008). However, in overdose, acetaminophen is hepatotoxic as it induces oxidative stress that subsequently causes mitochondrial impairment and hepatic necroptosis (Cranswick and Coghlan, 2000; Warwick, 2008). When acetaminophen is metabolized, the highly toxic acetaminophen metabolite N-acetyl-p-benzoquinone-imine is formed and gets conjugated to the hepatic store of reduced GSH. In case of acetaminophen overdose, N-acetyl-p-benzoquinone-imine reacts further with cellular proteins causing oxidative stress, lipid peroxidation, and excessive free radical production. Consequently, numerous studies in cells and animals have proven that oxidative stress plays an essential role in the toxic effects induced by acetaminophen (Wang et al., 2017). For example, 0.1 mM of acetaminophen decreased levels of cellular GSH and elevated levels of malondialdehyde, a highly reactive product from lipid peroxidation, in rat hepatocytes, while the antioxidant compound saponarin ameliorated acetaminophen-induced hepatoxicity by restoring GSH and malondialdehyde levels (Simeonova et al., 2013). Moreover, 6 mM of acetaminophen decreased GSH levels significantly and enhanced telomerase activity in rat embryonic liver cells (Bader et al., 2011). Even lower concentrations of acetaminophen, ranging from 0.05 to 0.3 mM, were found to increase ROS generation in mitochondria and induced the gene expression of NRF2, crucial for maintaining cellular redox homeostasis, in mouse hepatoma cells (Perez et al., 2011). Clinical trials revealed that acetaminophen treatment for more than 1 week decreases the antioxidative capacity in elderlies (Pujos-Guillot et al., 2012) and febrile children (Kozer et al., 2003). Moreover, a gradual decrease in serum antioxidant capacity, eventually by a reduction in GSH, was found in men and women after ingestion of maximum therapeutic doses of acetaminophen for 14 days (Nuttall et al., 2003). Combined administration of acetaminophen and N-acetyl-cysteine amide prevented the drop in levels of reduced GSH in liver mitochondria and reduced histopathologic hepatic lesions in C57BL/6 mice. Notably, the impact of N-acetylcysteine amide was superior to NAC, possible due to the derivative’s improved lipophilicity, membrane permeability, and antioxidant property (Khayyat et al., 2016). Moreover, recent studies also revealed hepatoprotective effects of the mitochondria-targeted antioxidant Mito-TEMPO in mice at late-stage acetaminophen overdose (Abdullah-Al-Shoeb et al., 2020), suggesting that scavenging of mtROS might be a promising approach to counteract acetaminophen-induced hepatotoxicity.

Acetylsalicylic acid, also known as aspirin and already introduced into the chapter “Antiplatelets”, is mainly used to reduce pain, fever, and inflammation. Moreover, acetylsalicylic acid is also specifically used to treat pericarditis, rheumatic fever, Kawasaki disease (Agarwal and Agrawal, 2017; Cortellini et al., 2017; Imazio et al., 2017). In addition to modulating the inflammatory response, acetylsalicylic acid affects the physiological function of platelets, counteracting the clotting. As discussed in the chapter “Antiplatelets”, low-dose acetylsalicylic acid is one of the most popular antiplatelet therapies for the treatment of patients with the acute coronary syndrome (Amsterdam et al., 2014), (Roffi et al., 20152016) and for the secondary prevention of atherothrombotic complications in high-risk patients (Parekh et al., 2013), (Patrono, 2015). Besides, several studies provided convincing evidence that regular low-dose acetylsalicylic acid use significantly lowers the risk of cancer (Rothwell et al., 2010; Algra and Rothwell, 2012; Chan et al., 2012; Ishikawa et al., 2013; Patrignani et al., 2017; Bosetti et al., 2020). Acetylsalicylic acid contains higher anti-inflammatory properties than acetaminophen (Mburu et al., 1990), probably because salicylic acid and its derivates also modulate signaling through NF-kB, which plays a crucial role in inflammation (McCarty and Block, 2006; Lawrence, 2009; Chen et al., 2018). It has also been suggested that aspirin converts COX-2 to lipoxygenase-like enzymes, which additionally results in the formation of mediators contributing to the anti-inflammatory effects of aspirin (Serhan and Chiang, 2013; Romano et al., 2015; Weylandt, 2016). Aspirin is the only non-steroidal anti-inflammatory drug (NSAID) not associated with increased cardiovascular events (Ghosh et al., 2015). Similar to acetaminophen and like the majority of NSAIDs, acetylsalicylic acid exerts its anti-inflammatory effects through inhibition of COX enzymes regulating the production of prostaglandins (Crofford, 1997). How acetylsalicylic acid influences ROS homeostasis seems partly unclear. Some studies show that aspirin decreases levels of ROS (Kim et al., 2017; Liu et al., 2019a; Liu et al., 2019b). For example, human hepatoma cells treated with 2- and 4-mM aspirin showed that aspirin remarkably decreased ROS levels (Liu et al., 2019a). Nucleus pulposus cells treated with 5 or 25 μg/ml aspirin significantly attenuated the production of NO and ROS (Liu et al., 2019b). Possibly, an application of rather low levels of aspirin or an application over short periods of time could eventually trigger *mitohormetic* responses (which is further discussed in chapter “Antiplatelets”) and thereby decrease ROS levels. Other studies propose an increase of ROS production in rat adipocytes, 1 µM of acetylsalicylic acid caused the activation of the NOX4 isoform of NADPH oxidase, boosting the generation of hydrogen peroxide (Vázquez-Meza et al., 2013). Moreover, 1 mM acetylsalicylic acid was found to increase lipid peroxidation in gastric small intestinal cells of rats (Nagano et al., 2012).

Opioids, which are often used as substitutes in the maintenance treatment for heroin addiction, modulate ROS homeostasis as well (Schiavone et al., 2019). Opioid use increases the production of ROS, which is suggested to play a role in opioid use disorders. It also leads to a decrease in the function of enzymatic antioxidants such as superoxide dismutase, catalase, and GPX and can elevate the risk of vitamin deficiency (Zahmatkesh et al., 2017; Salarian et al., 2018). Supplementation with antioxidants may help to restore the redox equilibrium. However, antioxidant therapy has not yet been proven to have a significant effect in randomized trials (Zahmatkesh et al., 2017).

### 4.6 Antibiotics

It has been demonstrated that major classes of bactericidal antibiotics, regardless of their molecular targets, trigger cell death in bacteria by acting as stressors leading to ROS overproduction (Dwyer et al., 2007; Kohanski et al., 2007; Wang et al., 2010b; Grant et al., 2012; Liu et al., 2012; Van Acker and Coenye, 2017). The mechanisms causing ROS overproduction involve the disruption of the TCA cycle and the ETC (Kohanski et al., 2007; Kohanski et al., 2008), as well as metabolism-related NADH depletion, damage of iron-sulfur clusters in proteins, and stimulation of the Fenton reaction (Dwyer et al., 2007; Kohanski et al., 2007; Van Acker and Coenye, 2017), mechanisms essential to maintain ROS homeostasis. Besides, studies suggested that low levels of ROS produced by sublethal levels of antibiotics might help bacteria to develop antibiotic resistance (Van Acker and Coenye, 2017; Rowe et al., 2020). Thereby, ROS might, for instance, trigger stress resistance mechanisms (Poole, 2012; Wu et al., 2012) or cause mutagenesis (Neeley and Essigmann, 2006; Kohanski et al., 2010; Jee et al., 2016; Van Acker and Coenye, 2017), helping bacteria to escape the bacteriocidic effect of antibiotics. According to the bacterial origin of mammalian cells’ mitochondria proposed by the endosymbiotic theory (Gray et al., 1999), it might be assumed that antibiotics target both pathogens and mitochondria of healthy cells. Indeed, bactericidal and bacteriostatic antibiotics have been shown to target mitochondrial components and function (Gootz et al., 1990; Hutchin and Cortopassi, 1994; McKee et al., 2006; Hobbie et al., 2008; Pochini et al., 2008; Lowes et al., 2009; Kalghatgi et al., 2013). For instance, chloramphenicol reversibly binds to the 50S subunit of the 70S ribosome in both prokaryotic organisms and mitochondria (Balbi, 2004), inhibiting peptidyl transferase, which catalyzes principal chemical reactions of protein synthesis. Align with this finding, tetracyclines like doxycycline and minocycline have been shown to impair mitochondrial biogenesis (Kroon and Van den Bogert, 1983), mitochondrial respiratory chain activity (Chatzispyrou et al., 2015), and mitochondrial protein synthesis (Fuentes-Retamal et al., 2020). Moreover, the macrolide antibiotic azithromycin has been shown to cause disruption of the *Ψ*_mito_ (Xiao et al., 2019), ROS production (Jiang et al., 2019), and cytochrome c release (Salimi et al., 2016).

Regardless of their specific molecular targets, three major classes of bactericidal antibiotics—quinolones, aminoglycosides, and β-lactams—have been associated with cause mitochondrial dysfunction, which leads to DNA-, protein-, and lipid damage, causing ROS-induced damage in mammalian cells (Kalghatgi et al., 2013). Consequently, oxidative cellular damage induced by bactericidal antibiotics may cause adverse side effects in humans after long-term use, including ototoxicity, nephrotoxicity, and tendinopathy (Brummett and Fox, 1989; Mingeot-Leclercq and Tulkens, 1999; Khaliq and Zhanel, 2003; Kalghatgi et al., 2013). Patients with weakened antioxidant defense systems or people genetically disposed to developing a mitochondrial dysfunction disease (Schaefer et al., 2008) may be at higher risk from bactericidal antibiotic treatments. Notably, co-administration of bactericidal antibiotics and NAC reduced side effects without reducing the bacterial killing efficacy of antibiotics (Kalghatgi et al., 2013). Besides, bacteriostatic antibiotics, such as tetracycline, did not contribute to the overwhelming production of ROS in mammalian cells (Kohanski et al., 2007) and showed fewer side effects (Kalghatgi et al., 2013). Notably, it has been proposed that the usage of specific antibiotics such as tetracyclines might be even beneficial due to the generation of mild mitochondrial stress that leads to activation of the mitochondrial unfolded protein response and enhanced stress resistance (Suárez-Rivero et al., 2021). In addition, doxycycline was found to promote fitness and survival in a Leigh syndrome mouse model and to rescue cell death and inflammatory signatures in cells carrying mitochondrial mutations by inducing a *mitohormetic* response (Perry et al., 2021). In summary, it seems crucial to which extent antibiotics cause ROS production and whether treated individuals exhibit a functional antioxidant defense system.

## 5 Conclusion

**FIGURE 1:**
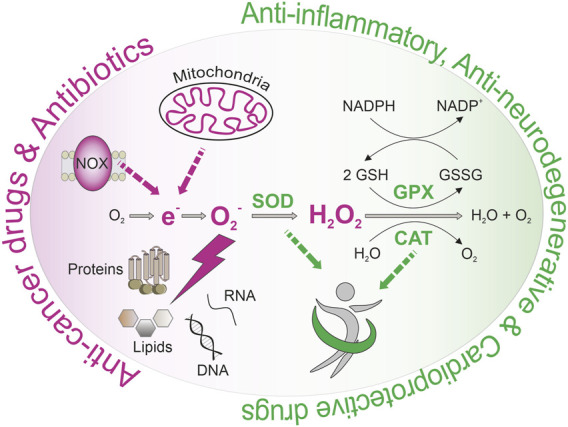
Intracellular ROS homeostasis affecting health and disease.

The current review provides an overview of the implication of ROS in pathological dysregulations and age-related diseases and the mechanisms of how approved drugs modulate ROS homeostasis (Figure 1). Drug classes of antibiotics and anti-cancer agents induce overwhelming ROS production and might thereby help to trigger the death of pathogens or cancer cells. However, thereby may also harm healthy cells. In contrast, drugs against diabetes, neurodegeneration, and CVD, as well as anti-inflammatory compounds, are often associated with boosting antioxidant defense mechanisms and thus preventing ROS-mediated damage of DNA, RNA, lipids, and proteins. In addition, the review highlights the potential of repurposing drugs against metabolic diseases for the treatment of neurodegenerative diseases. For most described drugs, it remains to be questionable whether ROS manipulation is a desirable side-effect or suitable as a drug’s primary mode of action. Moreover, repurposing drugs in use might help to spare time in clinical trials since safety is already confirmed, but specific targeting of ROS sources or detoxification sites might be necessary to enhance the effect or avoid multiple targets and, thereby, side-effects. The individual ROS homeostasis and antioxidant potential undergo crucial alterations during aging (Beal, 2002; Suh et al., 2003; Choksi et al., 2008). Consequently, it might be essential to determine the proper intervention time and adjust the dosage of drugs dependent on the individual oxidative state. Besides, it still has to be clarified which intensity and duration of ROS signals are suitable to induce long-lasting effects in signaling. Moreover, fundamental questions must be solved, including determining the intensity and duration of ROS modulation to cause a long-lasting impact. Thereby, live-cell imaging methods enabling the real-time tracking of various ROS species in different cellular organelles are essential, and reliable blood markers for ROS generation and detoxification might have to be characterized to monitor patients. Manipulating ROS might be a promising strategy to induce toxic effects against pathogens or cancer cells. In addition, specific modulation of ROS levels during aging might be utilized to enhance defense mechanisms and, thereby, prevent the development and progression of cardiovascular and neurodegenerative diseases.
